# *In silico* models of the macromolecular Na_V_1.5-K_IR_2.1 complex

**DOI:** 10.3389/fphys.2024.1362964

**Published:** 2024-02-26

**Authors:** Anna Stary-Weinzinger

**Affiliations:** Division of Pharmacology and Toxicology, Department of Pharmaceutical Sciences, University of Vienna, Vienna, Austria

**Keywords:** Nav1.5, KIR2.1, protein-protein interactions, disease hotspot, trafficking, channelosomes

## Abstract

In cardiac cells, the expression of the cardiac voltage-gated Na^+^ channel (Na_V_1.5) is reciprocally regulated with the inward rectifying K^+^ channel (K_IR_2.1). These channels can form macromolecular complexes that pre-assemble early during forward trafficking (transport to the cell membrane). In this study, we present *in silico* 3D models of Na_V_1.5-K_IR_2.1, generated by rigid-body protein-protein docking programs and deep learning-based AlphaFold-Multimer software. Modeling revealed that the two channels could physically interact with each other along the entire transmembrane region. Structural mapping of disease-associated mutations revealed a hotspot at this interface with several trafficking-deficient variants in close proximity. Thus, examining the role of disease-causing variants is important not only in isolated channels but also in the context of macromolecular complexes. These findings may contribute to a better understanding of the life-threatening cardiovascular diseases underlying K_IR_2.1 and Na_V_1.5 malfunctions.

## 1 Introduction

Milstein et al. showed that the expression of the cardiac voltage-gated Na^+^ channel (Na_V_1.5, encoded by the *SCNA5* gene, I_Na_ current) is reciprocally regulated by the inward rectifying K^+^ channels (K_IR_2.1, encoded by the *KCNJ2* gene, I_K1_ current) in cardiac cells (Milstein et al., 2012). Both ion channels play key roles in cardiac excitability. Rapid depolarization of the membrane potential is mediated by Na_V_1.5, which allows an influx of positively charged Na^+^ ions, resulting in a rapid upstroke of the cardiac action potential. On the other hand, K_IR_2.1 is responsible for shaping the initial depolarization, final repolarization, and resting phases of the ventricular action potential (Ibarra et al., 1991). Consequently, these two channels are the most important ionic currents that control ventricular excitability. The essential role of both ion channels is further emphasized by the fact that dysfunction is associated with a high risk of arrhythmias and sudden cardiac death (Abriel, 2007; Cui et al., 2021; Reilly and Eckhardt, 2021; Hager et al., 2022).

The functional interaction between *I*_K1_ and *I*_Na_ is indirectly mediated via voltage dependence; however, increasing evidence suggests a direct interaction between these two channels. Moreover, K_IR_2.1 expression has been shown to increase the expression of Na_V_1.5 channels and *vice versa* (Milstein et al., 2012; Utrilla et al., 2017). A study by Ponce-Balbuena et al. (2018) revealed that the macromolecular complex Na_V_1.5-K_IR_2.1 preassembles early in the forward trafficking pathway, with important implications for the mechanisms of inherited arrhythmias. Using a trafficking-deficient K_IR_2.1 mutant that causes the rare channelopathy Andersen-Tawil Syndrome (ATS1), the authors revealed mutual impairment in the forward trafficking of wild-type (WT) Na_V_1.5 channels. The potential reduction in I_Na_ due to the reduction in I_K1_ leads to the breakdown of the macromolecular complex between the two channels and contributes to the susceptibility of patients with ATS1 (Moreno-Manuel et al., 2023). Similarly, the trafficking of defective Na_V_1.5 mutants that leads to the rare Brugada syndrome (BrS) can negatively affect I_K1_ by trapping cardiac K_IR_2.1/2.2 channels, contributing to the pathogenesis of arrhythmias (Pérez-Hernández et al., 2018). While an increasing number of studies have underscored the physiological importance of the Na_V_1.5-K_IR_2.1 complex, very little is known about the precise molecular nature of the macromolecular complex. CaMKII inhibition precludes the interaction between Na_v_1.5 and K_IR_2.1 channels (Pérez-Hernández et al., 2018). Furthermore, inhibition of the 14-3-3 proteins reduces the co-expression of I_Na_ and I_K1_ currents without abolishing their interaction. Proximity ligation assay studies revealed the close proximity of Na_V_1.5 and K_IR_2.1 proteins in the membrane of ventricular myocytes (<40 nm apart) (Utrilla et al., 2017). Furthermore, both ion channels have been shown to co-immunoprecipitate in forward and reverse co-immunoprecipitations, emphasizing their direct interaction. However, only a subset of channels is presumed to co-assemble into macromolecular complexes known as channelosomes (Milstein et al., 2012; Ponce-Balbuena et al., 2018). Despite extensive analyses of the physiological importance of macromolecular complexes (for a recent review, see (Reilly and Eckhardt, 2021), structural insights into the Na_V_1.5-K_IR_2.1 macromolecular complex are still lacking. The 3D structures of isolated human K_IR_2.1 (Fernandes et al., 2022) and human Na_V_1.5, channels (Jiang et al., 2020; Li et al., 2021) were solved at the atomic level using cryo-electron microscopy (cryo-EM). Na_V_1.5 channels consist of four homologous transmembrane domains (DI–DIV) linked by three intracellular loops containing six segments (S1–S6). Cryo-EM structures provide detailed insights into the voltage-dependent activation, inactivation gates, ion selectivity filters, and drug blocks of several antiarrhythmic drugs (Noreng et al., 2021). Only one cryo-EM structure of the human K_IR_2.1 channel has been published in a closed conformation (Fernandes et al., 2022). K_IR_2.1, which can form homotetrameric and heterotetrameric pores, consists of a canonical pore-forming transmembrane domain (TMD) with two transmembrane helices (M1 and M2) separated by a K^+^ selectivity filter, and a large cytoplasmic domain (CTD) containing N- and C-termini (Hibino et al., 2010).

These atomic-resolution structures make it possible to use computational methods to investigate the physical interactions between the two channels. In this study, we report *in silico* 3D models of macromolecular Na_V_1.5-K_IR_2.1 complexes, generated by rigid-body protein-protein docking programs and a deep learning-based AlphaFold-Multimer.

## 2 Results and discussion

### 2.1 Close physical interactions between Na_V_1.5 and K_IR_2.1 in the closed conformation

The prediction of protein-protein interactions (PPIs) is a challenging process, both experimentally and computationally, and the success rates of these computational methods are still relatively low (Vreven et al., 2015). Considering the limitations and challenges of computational methods (for a recent review, see (Vakser, 2020), we combined rigid-body docking strategies and deep learning-based methods for obtaining the most realistically possible insights into the macromolecular Na_V_1.5-K_IR_2.1 complex. Three *in silico* programs were selected based on their ability to handle large proteins. As detailed in the Methods section, two rigid-body docking programs, FRODOCK (Garzon et al., 2009), ClusPro (Kozakov et al., 2017), and the AlphaFold-Multimer (v3) deep learning tool (Evans et al., 2021), trained for PPI, were used to predict 3D interactions between Na_V_1.5 and K_IR_2.1.

Based on the experimental finding that channels assemble early during forward trafficking (Ponce-Balbuena et al., 2018), the 3D coordinates of the channels in closed conformations were chosen as the starting points for modeling. This is based on the assumption that in order to transiently open, Na_V_ channels require depolarizing conditions, not present during forward trafficking. Further, all experimental 3D structures of eukaryotic K_IR_ channels have been obtained in closed state conformations, unless gating modifying mutations have been introduced, suggesting that this state is energetically favored and might thus represent a plausible trafficking conformation.

The cryo-EM structure of the full-length human Na_V_1.5 channel was obtained at 0 mV, with the voltage sensor modules in the “up-state,” and the intracellular gate in a constricted, presumably inactivated state, inhibited by quinidine (Li et al., 2021). The cryo-EM structure of the human K_IR_2.1 channel represents a fully closed state with the cytoplasmic domain disengaged from the transmembrane domain and the helix bundle-crossing region restricted by two hydrophobic residues (Fernandes et al., 2022).

**FIGURE 1:**
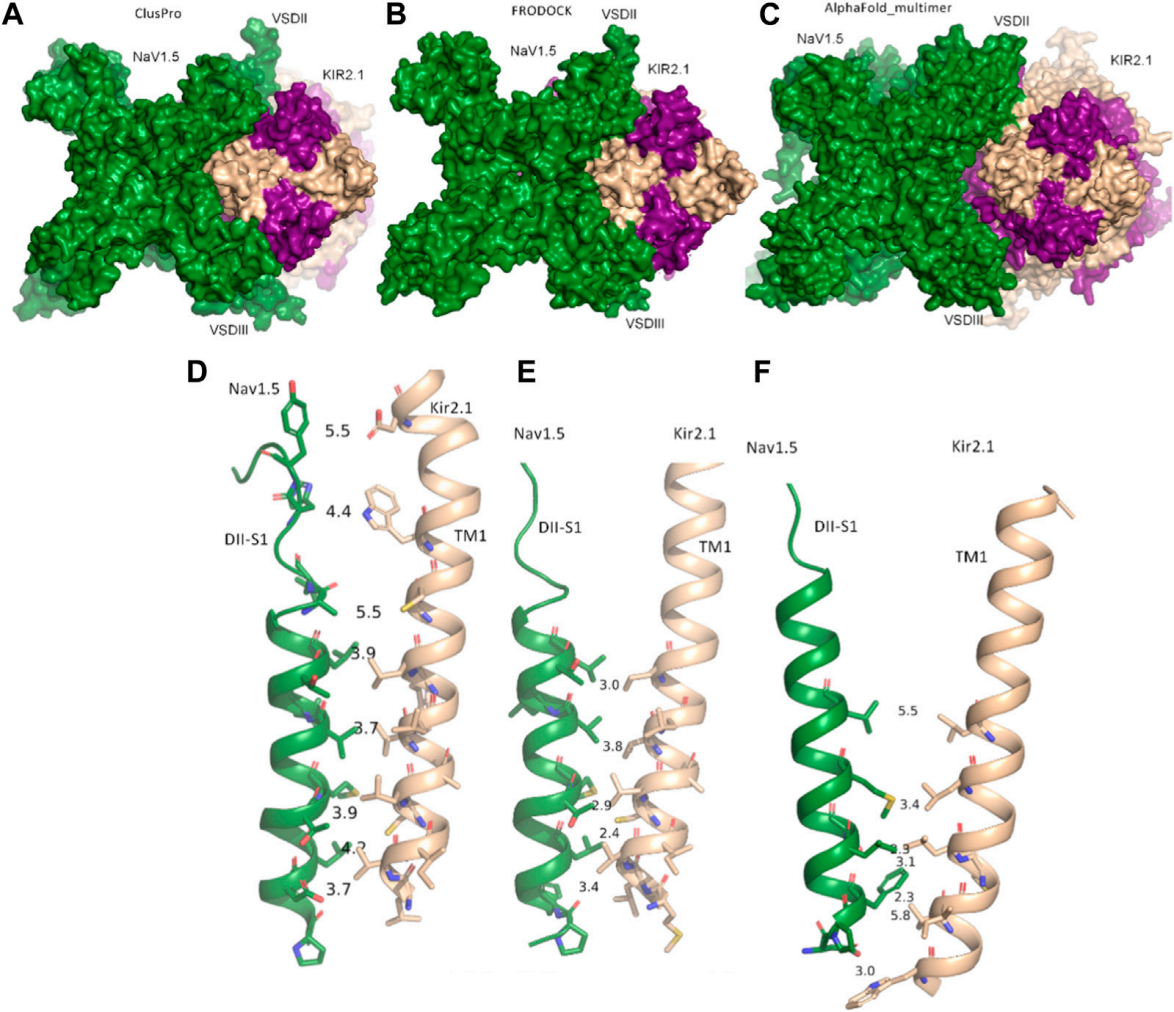
Modeled interactions between Na_V_1.5 and K_IR_2.1. **(A)** Surface representation of the best predicted Na_V_1.5-K_IR_2.1 complex from ClusPro in top view. **(B)** Surface representation of the best-predicted Na_V_1.5-K_IR_2.1 complex from FRODOCK in top view. **(C)** Surface representation of the best-predicted Na_V_1.5-K_IR_2.1 complex from AlphaFold-Multimer in top view. **(D–F)** Consensus interface between helices DII-S1 from the VSD of Na_V_1.5 is shown as a green drawing with residues containing atoms within 6 Å shown as sticks. The distances between the selected residue pairs are shown. D, predicted interactions from ClusPro; E, predicted interactions from FRODOCK; and F, predicted interactions from AlphaFold-Multimer. Na_V_1.5 is shown in green, while two subunits of K_IR_2.1 are colored in light orange, and the opposing subunits are colored in violet.

Both rigid-body docking programs predicted a favorable interface between the two channels over the entire transmembrane region (Figures 1A,B).

Extensive interactions were observed between helices S3 and S4 of VSD-III, helices III-S5, and the outer P-helical segment of domain III (Figures 1A, B; Supplementary Figure S1A). The overall interface area was large, with values of up to 3652.4 Å^2^ (see Table 1). Most intermolecular contacts between Na_V_1.5 and K_IR_2.1 were hydrophobic in nature. This was predictable because the majority of the interface lies in the membrane-spanning region. In addition, 21 hydrophilic contacts (hydrogen bonds and salt bridges) were identified in the best model obtained using ClusPro (Table 1; Supplementary Table S1. Interface residues within 5.5 Å predicted by FRODOCK are listed in Supplementary Table S2. Extensive contacts between Na_V_1.5 and K_IR_2.1 might also be formed in the cytoplasmic domain. Unfortunately, these regions, particularly in the Na_V_1.5 channel, lack a cryo-EM structure and cannot be reliably modeled with AlphaFold (see Supplementary Figure S3), precluding further analysis of putative intracellular contacts.

**TABLE 1:** Interface analysis; abbreviations: C, charged; P, polar; A, apolar.

	PISA-PDBe				Prodigy		
Model	Interface area Å^2^	ΔG Kcal/mol^-1^	Hydrogen bonds	Salt bridges	ΔG Kcal/mol^-1^	Intermolecular contacts C-C/C-P/P-P/P-A/A-A	Non-interacting surface (%) C/A
ClusPro2.0	3594.9	−89.1	16	6	−15.2	9/15/34/2/33/137	22.67/48.02
FRODOCK	3652.4	−57.6	2	1	−14.5	2/5/22/1/40/157	22.4/51.33
AlphaFold-Multimer v3	457.7	−8.7	1	0	−5.9	1/1/12/0/3/14	24.82/46.22

### 2.2 State-dependent changes in the Na_V_1.5 - K_IR_2.1 interface

**FIGURE 2:**
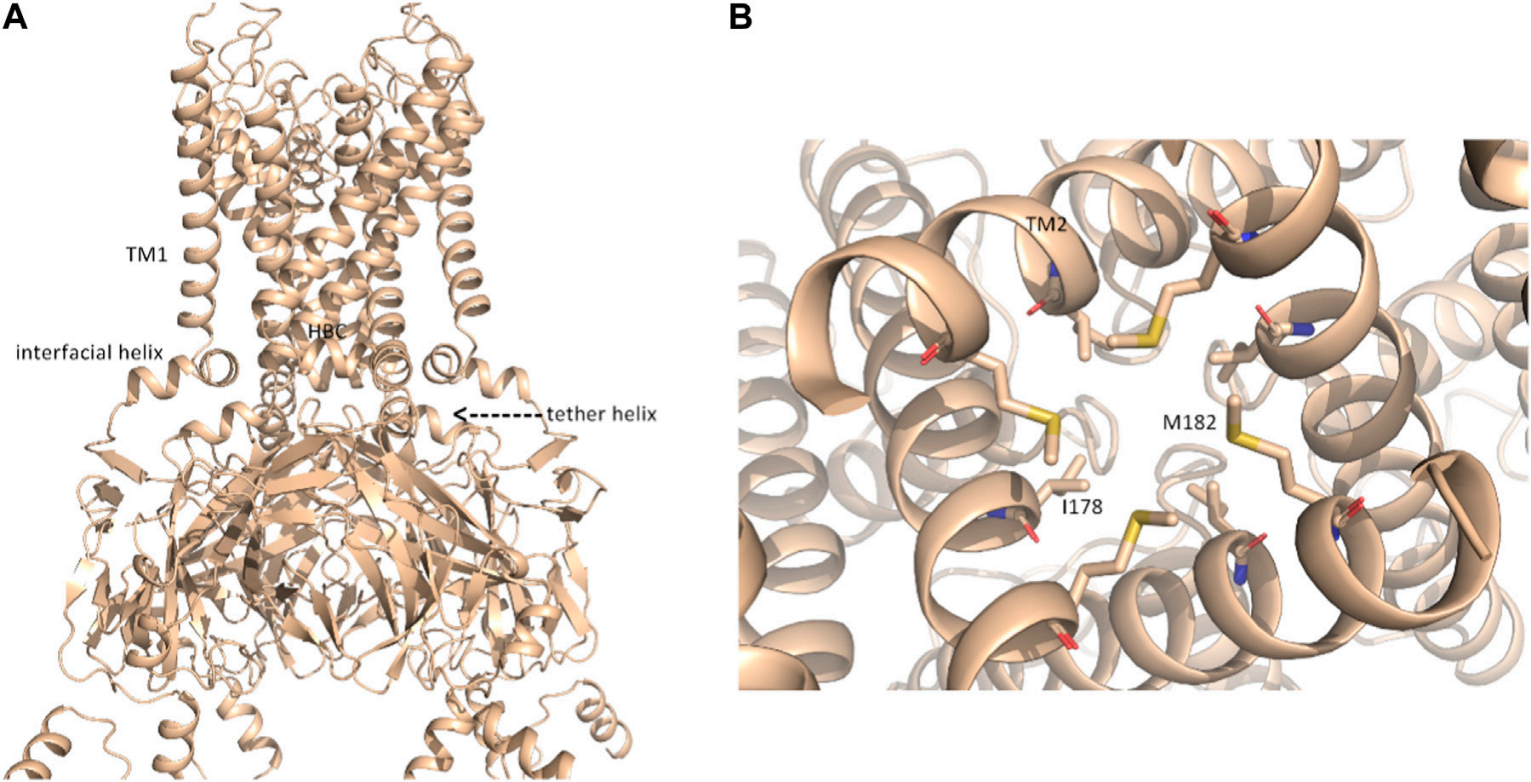
putative open-state model of K_IR_2.1. **(A)** Representation of the open-state K_IR_2.1 model from AlphaFold in side view; **(B)**, magnification of the HBC gate, viewed from the bottom, with closed-state constricting residues I178 and M182 shown as sticks.

In contrast to rigid docking programs, in which the conformation of the interaction partners can be actively chosen, the conformations modeled by AlphaFold-Multimer cannot be selected *a priori*. The AlphaFold-Multimer consistently models the K^+^ channel in the open-channel state, with an engaged cytoplasmic domain (Figures 2A, B; Supplementary Figure S1B). Supplementary Figure S1A show the structural superposition of Na_V_1.5 and K_IR_2.1 channels with their respective cryo-EM structures.

Although the AlphaFold Multimer was run in ‘no template mode’, it modeled the conformation of K_IR_2.1, highly similar to the open, conductive state of K_IR_2.2 (Zangerl-Plessl et al., 2019) with an overall RMSD of 1.47 Å between the two structures (see Supplementary Figure S1C). In the modeled structure, both the interfacial and tether helices folded, and the hydrophobic residues I178 and M182 did not restrict ion flux through the HBC gate, as seen in the closed cryo-EM state (Figure 2B). This was unexpected given the lack of the main activatory ligand PIP_2_ or anionic lipids, which are crucial for channel activation gating (Hibino et al., 2010; Lee et al., 2016).

In this gating state, the predicted interface between Na_V_1.5 and K_IR_2.1 was much smaller, with a surface area of 457.7 2 Å (Table 1). This suggests that PPI between the two channels is highly sensitive to their gating states. Remarkably, however, a favorable interface between the voltage sensor domains (VSDs) of domain II and the transmembrane helix 1 (TM1) of the K_IR_2.1 channel was observed in both the rigid-body closed-state complex and AlphaFold-predicted open-state models (Figures 1C,F), suggesting state-independence of this interface, when considering open vs. closed K_IR_2.1 states. The movement of TM1 during K_IR_2.1 channel gating is negligible, as can be seen, when comparing the closed and open state structure models respectively (Supplementary Figure S1B). The situation is less clear for voltage sensor movement of Na_V_1.5. Our knowledge about VSD movement in Na_V_ channels is far from complete, but a cryo-EM-structure of a toxin induced deactivated state of a Na_V_1.7 VSD with a resolution of 4.2 Å is available (Xu et al., 2019). This structure suggests that helices S1-S3 undergo rigid body shifts of ∼ 3Å, while the S4 helix translates ∼ 10 Å during gating. Assuming that similar structural arrangements occur during normal electromechanical coupling in Na_V_1.5 voltage sensor deactivation would influence KIR2.1 interaction. A more detailed understanding will require extensive further studies, beyond the scope of the current work.

As shown in Figures 1D–F, the interactions at this interface were hydrophobic. The AlphaFold model is colored according to the per-residue model confidence score (pLDDT), which provides a measure of the confidence of the respective residue (see Supplementary Figure S3 for the complete structure). All residues at the interface (DII-S1 helix) had high to very high confidence scores.

While, the region of Na_V_1.5 predicted to interact with K_IR_2.1 was modelled in a state very similar to

**FIGURE 3:**
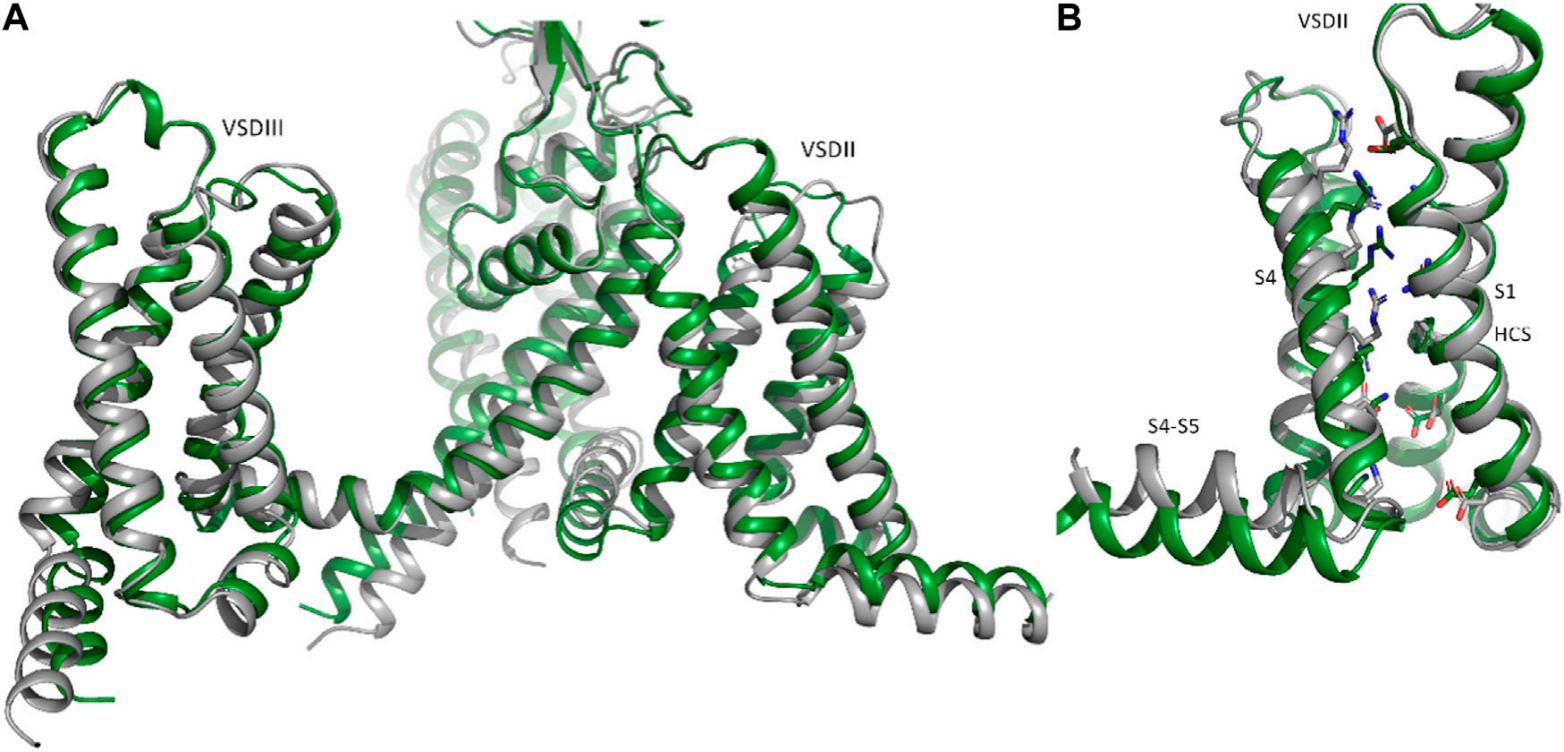
Comparison of Nav1.5 cryo-EM and Alphafold structures. Identification of putative disease hotspots at the DII_S1- TM1 interface. **(A)** Representation of domains II and III of the Na_V_1.5 AlphaFold model in side view; **(B)**, isolated voltage sensor of domain II, with the charge carrying residues in S4, conserved acidic residues and the hydrophobic constriction site (HCS) are shown as sticks.

The available cryo-EM structure, with an overall RMSD of 1.45 Å between the two structures (Figure 3 A, B). However, deviations at the intracellular side of the transmembrane region (e.g., DII-S4-S5 helix, as shown in Figure 3B) are observed. The voltage sensor of domain II is predicted in the “up-state” in both the cryo-EM structure and the Alphafold model, however R814 is positioned slightly below the hydrophobic constriction site (HCS) in the latter. While the pLDDT score in helix DII-S4 is indicating high model quality (>70), this is not the case for DII-S4-S5, with low pLDDT scores (∼65), indicating low reliability. Thus, these deviations should not be interpreted. Of note, the low model quality at the intracellular half of the transmembrane region and in particular of large parts of the intracellular regions do not result from the dimerization ‘experiment’, but are also seen in the isolated Na_V_1.5 model, available from the Alphafold database. The reason for the low pLDDT scores are unclear, but are reflected in the missing cryo-EM densities in these regions.

### 2.3 Trafficking-deficient variant hotspots at the Na_V_1.5-K_IR_2.1 interface

Given the experimentally documented relevance of disease-linked mutations to the function of the macromolecular complex (Ponce-Balbuena et al., 2018), structural mapping of disease-associated missense and deletion mutations was performed. Table 2 provides an overview of all missense variations identified in the DII-S1-TM1 interface. As shown in Figure 3, six missense mutations (A735 V/E and T731I in Nav1.5; C101R, V93I, L94P, and R82W/Q) and one deletion (del S95-F98) were positioned in the predicted binding interface. In particular, the C101R mutation led to trafficking defects (Ballester et al., 2006). Clinically, this mutation presents with a phenotype of life-threatening events associated with polymorphic ventricular tachycardia (Chun et al., 2004), which requires treatment with an implantable cardioverter-defibrillator. Since previous work by the Jalife lab (Ponce-Balbuena et al., 2018) revealed that K_IR_2.1 mutants could negatively influence Na_V_1.5, experimentally testing this effect for C101R is essential. Structurally, arginine side chains possibly play a disruptive role at the protein-protein interface. Similarly, L94P has been shown to negatively affect plasma membrane trafficking, leading to a reduction in the number of functional channels on the cell surface (Takeda et al., 2013). Co-expression with wild-type subunits rescued channel trafficking, suggesting a non-dominant effect of this mutation. Similarly, an in-frame deletion, del S95-F98, has been shown to negatively impact K_IR_2.1 trafficking; however, it remains trapped in the cytoplasmic domain, even when co-expressed with the wild-type (Plaster et al., 2001). The deletion of four helical residues in TM1 clearly poses far-reaching effects on the secondary structure and consequently on the putative channel-channel interface.

**TABLE 2:** details of disease associated hotspot mutations.

Diseasevariant	Channel	Allelic variation	Phenotype	Functional data	References
A735V	Na_V_1.5	No data	BrS	shifts activation curve to the right, possibly decelerates recovery from inactivation	Vatta (2002)
					De La Roche et al. (2019)
A735E	Na_V_1.5	No data	BrS, cardio-pulmonary arrest, sinus node dys-function, exercise and epinephrine-induced QT prolongation, supraventricular tachycardia	Loss-of-function	Glazer et al. (2020), Sasaki et al., 2021
T731I	Na_V_1.5	No data	long QT3 syndrome and an increased risk of arrhythmias	No data	Kapplinger et al. (2009)
					Pan et al. (2021)
C101R	K_IR_2.1	heterozygous, dominant negative	polymorphic ventricular tachycardia	trafficking-defective; complete loss of channel function, when expressed in oocytes	Chun et al. (2004)
					Ballester et al. (2006)
Del S95-F98	K_IR_2.1	No data	ATS1	Trafficking defective, dominant negative; impaired protein folding	Plaster et al. (2001)
L94P	K_IR_2.1	homozygous mutation	ATS1	Trafficking defective, can be rescued, when co-expressing with wild-type subunits	Takeda et al. (2013)
V93I	K_IR_2.1	heterozygous	familial atrial fibrillation, long QT syndrome	Gain of function	Xia et al. (2005)
					Zaklyazminskaya et al. (2022)
R82W	K_IR_2.1	heterozygous, dominant negative effect	ATS1	PIP_2_-dependent gating regulation	Eckhardt et al. (2007)
R82Q	K_IR_2.1	heterozygous, dominant negative effect	ATS1	PIP_2_-dependent gating regulation	Eckhardt et al. (2007)

Mutation V93I is associated with familial atrial fibrillation and can lead to increased channel activity (Xia et al., 2005). *In vitro* analysis of channel function revealed a gain-of-function for this variant (Xia et al., 2005; Zaklyazminskaya et al., 2022). A study of Russian patients with this variant revealed a surprising phenotype without any signs of ATS1 or mild but evident QTc prolongation (Zaklyazminskaya et al., 2022). Thus, the clinical significance of this variant remains unclear; however, it is classified as a potential proarrhythmogenic risk factor. A structural interpretation based on the putative Na_V_1.5-K_IR_2.1 complex suggests increased stability of the interface due to V93I, which fits the experimentally observed increase in channel activity. Explaining the reason behind the alterations in channel function following replacement of the valine side chain with the bulkier isoleucine side chain, based on the isolated K_IR_2.1 structure, remains challenging. In the non-complexed state, this residue faces toward the lipid membrane, and unless currently unknown specific lipid interactions occur at this site, the impact of isoleucine should be negligible. Further, position V93 is not a highly conserved residue, and several species possess isoleucine at that position (Houtman et al., 2014). Further studies on the importance of this putative disease variant are required to clarify its role.

The missense variants of R82W/Q have been implicated in PIP_2_-dependent gating regulation (Eckhardt et al., 2007). Thus, mechanical dissection of mutant effects requires structural determination of the channel complex in multiple functional states. This requires thorough experimental validation of the predicted complex, which is beyond the scope of the current study.

The missense variant A735V in Na_V_1.5 has been reported to shift the activation curve to the right and possibly decelerate recovery from inactivation. No obvious changes in channel expression or membrane insertion were observed. Furthermore, this mutant has been shown to reduce the upstroke velocity of action potentials in hiPSC-CMs (De La Roche et al., 2019). Phenotypically, the A735V mutation is associated with Brugada syndrome and can cause malignant arrhythmias and ventricular fibrillation, leading to sudden cardiac death (Vatta, 2002). On the contrary, the phenotypic manifestation of the A735E mutant is very complex, poorly understood, and difficult to explain, when considering only the effects of Na_V_1.5 (Sasaki et al., 2021). Thus, Sasaki et al. (2021) suggested that this mutation might affect other functionally or structurally associated proteins. Na_V_1.5 interacts with a multitude of different auxiliary proteins, associated proteins, anchoring proteins, β-subunits, scaffolding proteins, adapter proteins, and regulatory proteins, a group of which harbor disease-causing mutations that have been shown to affect I_Na_ (Kyle and Makielski, 2014). Thus, the K_IR_2.1 interaction may be speculated to play a crucial role here. However, functional studies in this area are lacking. Therefore, testing for disease mutations may be informative in the context of the macromolecular Na_V_1.5-K_IR_2.1 complex. An interesting hypothesis derived from the modeled complex suggests that the formation of a salt bridge between disease variants A735E and C101R might be possible. This could potentially stabilize the trafficking of C101R and positively influence the missense variant A735E. Such analyses would not only help to test and validate the predicted interface, but also provide crucial information on the complex mechanism of inherited cardiac diseases resulting from defects in Na_V_1.5 and K_IR_2.1. Mutation T731I is associated with Long QT3 syndrome and an increased risk of arrhythmias (Pan et al., 2021). However, no further details regarding this mutation are available in the literature.

In summary, three of four ATS1 mutations predicted interface-induced trafficking defects. This is interesting because experimental studies have revealed the importance of the macromolecular complex for trafficking (Ponce-Balbuena et al., 2018). Since the impairment of K_IR_2.1 trafficking in cardiomyocytes affects Na_V_1.5 trafficking and *vice versa*, studying the mechanisms of these inheritable disease mutants is crucial in the context of both interaction partners.

### 2.4 Functional implications resulting from the proposed Na_V_1.5-K_IR_2.1 model

Modeling predictions suggest that the main role of the macromolecular Na_V_1.5-K_IR_2.1 complex is to stabilize during forward trafficking, interacting with a large interface as the complex pre-assembles early (Ponce-Balbuena et al., 2018). In line with this, the disruption of trafficking by one partner has been shown to affect trafficking by the other. However, both channels interact through common partners, such as scaffolding, anchoring, adaptor, and regulatory proteins (Park et al., 2020), whose atomic interactions cannot be reliably modeled with current programs.

Evidence from experimental studies suggest that a subpopulation of Na_V_1.5-K_IR_2.1 channels can physically interact when bound to the plasma membrane. First, both channels travel through similar retrograde trafficking (endosome-to-Golgi transport) routes. Additionally, co-expression of the two channels resulted in an increase in Na_V_1.5 density. Moreover, both channels have been shown to co-immunoprecipitate in both the forward and reverse co-immunoprecipitation reactions (Ponce-Balbuena et al., 2018). Independent studies by Eckhardt and Jalife have demonstrated that K_IR_2.1 and Na_V_1.5, colocalize in ventricular myocytes in humans, rats, and mice (Milstein et al., 2012; Vaidyanathan et al., 2018).

Based on the predicted physical interaction models presented here, the gating dynamics of both channels clearly influence physical interactions. Relevant interface changes need to be addressed in future experimental studies, such as crosslinking, to confirm the predicted tight physical interaction of the macromolecular Na_V_1.5-K_IR_2.1 complex at the plasma membrane.

Tighter control of the resting membrane potential and excitability in the ventricles may be possible through strictly and simultaneously regulating the expression of these two channels and their physical contact. This is beneficial in preventing fibrillating arrhythmias (Utrilla et al., 2017). Given the close contact between the voltage domains of Na_V_1.5 and TM1 of K_IR_2.1 (Figures 1D–F), voltage-dependent structural changes from the VSD of Na_V_1.5 might also be transmitted to the K_IR_2.1 pore. Sigg, Chang, and Shieh (Sigg et al., 2018) suggested the presence of an unknown voltage sensor in K_IR_2.1, when studied in the absence of native intracellular blockers, such as Mg^2+^ and polyamines. Their study suggested that an unknown voltage sensor communicating allosterically with the pore gate best explains the weak inward rectification in the K_IR_2.1 channels observed. Furthermore, the authors propose that this voltage sensor stabilizes the closure of the pore gate with a coupling factor of ∼31. Based on the current modeling predictions, it is tempting to speculate that the unknown voltage sensor might actually be located on Na_V_1.5. This hypothesis would require *Xenopus laevis* oocytes, which were used as the measuring system, to contain native sodium currents, similar enough to form macromolecular complexes with the injected K_IR_2.1 channels. A BLAST search of the human Na_V_1.5 channel identified a protein sequence in *Xenopus laevis* with approximately 70% sequence identity and almost 80% sequence similarity. However, further studies are required to test this hypothesis.

A recent high-speed atomic force microscopy study of the bacterial voltage-dependent Na_v_Ab channel revealed that VSDs can dissociate from the pore in the resting state and dimerize to form cross-links between channels (Sumino et al., 2023). In the proposed tight interaction model between Na_V_1.5 and K_IR_2.1, the dissociation of the VSDs is unlikely. It can be expected, that the dynamics of the predicted Na_V_1.5-K_IR_2.1 complex are different from those of the individual isolated channels.

While most experimentally resolved structures of Na_V_ channels display voltage sensors in the “up-state” (activated), a recent 2.7-Å resolution Na_V_1.7 structure was captured with one VSD in a completely deactivated (down) state. Voltage-dependent structural changes involve a combination of helix unwinding and spiral sliding of helix S4, and a rotation of the entire VSD domain of approximately 20° (Huang et al., 2022). Although S4, is not involved in the PPI, and movements of S4 are unlikely to influence the Na_V_1.5-K_IR_2.1 interface (Figure 1), rotation of the whole VSD could have a significant impact.

Nature must have designed this subpopulation of Na_V_1.5-K_IR_2.1 channelosomes for some unique biological advantage(s); however, much research is needed to fully understand their specific biological roles and differences to potentially oligomerized Na_V_1.5 channels (reviewed in (Iamshanova et al., 2023).

Interestingly, sodium ions directly affect K_IR_2.1 function. External Na^+^ acts as a competitive inhibitor of K^+^ conductance through K_IR_2.1, which is physiologically relevant under certain pathological conditions. Specifically, this may increase outward K^+^ efflux through K_IR_2 channels under medical conditions, concomitant with severe hyponatremia (Ishihara, 2018).

In summary, the current study revealed that tight physical interactions are likely. In fact, modeling predictions suggest a large interface area (>3600 Å^2^) with a favorable “binding energy,” consistent with other PPIs. More importantly, the interface was independently predicted using three different programs, supporting the validity of the prediction. Finally, atomic insights into the Na_V_1.5-K_IR_2.1 complex enable interesting structural interpretations of disease-causing missense variants in both channels. In fact, mutations, such as V93I (K_IR_2.1, Figure 3), are difficult to rationalize in the absence of a tightly bound Na_V_1.5 channel.

### 2.5 Limitations of the study

**FIGURE 4:**
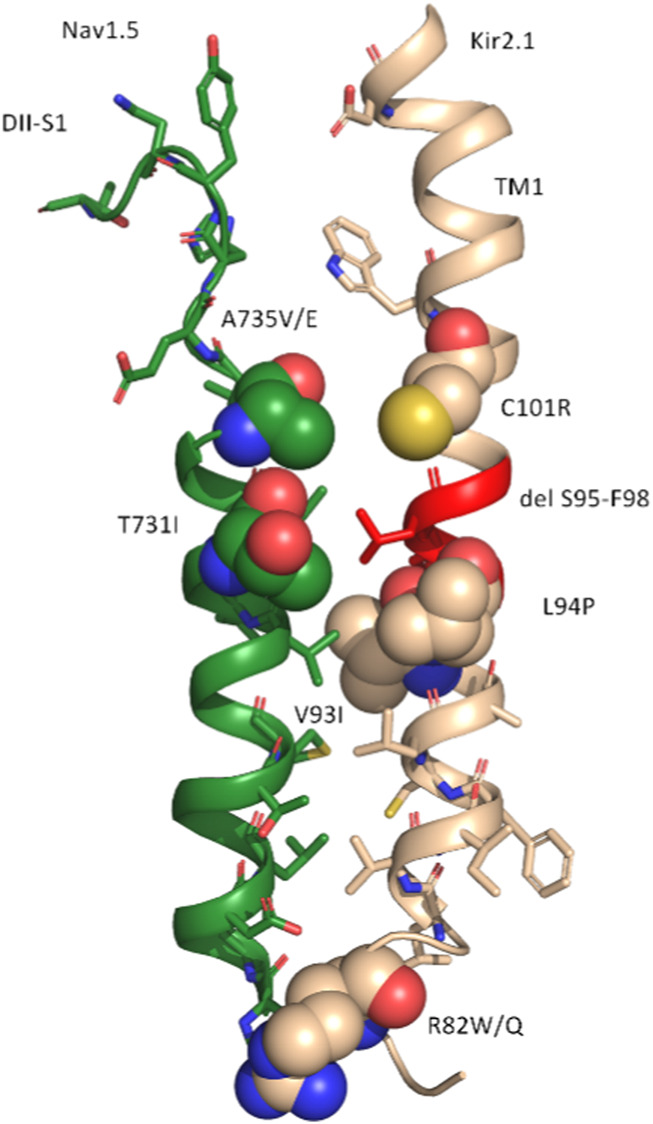
Representation of the consensus interface between DII-S1 (Na_V_1.5) and TM1 (K_IR_2.1) of the ClusPro model. All residues with atoms within 6 Å of the interface are shown as sticks. Disease-linked missense variants are shown as spheres, colored in green for Na_V_1.5 and in wheat for K_IR_2.1. The disease-linked deletion delS95-F98 in Kir2.1 is colored in red.

Further studies are required in this exciting area of research. The validity of the *in silico* model needs to be thoroughly tested. The PPI models presented herein have not been refined, which would optimally require extensive molecular dynamics (MD) simulations, which will be done in future studies. Further, Figure 4 highlights a disease hotspot, but the mutants have not been modeled and their interactions investigated. This also would require MD refinement of the PPIs and preferably extensive side-chain resampling by simulations, which is beyond the scope of the current study. Further, experiment validation is necessary, for example, via cross-linking studies, for which the current study provides crucial starting points. The current state-of-the-art computational protein-protein interaction methods still perform rather poorly in predicting large macromolecular protein complexes. One particular limitation is the lack of 3D coordinates for large intracellular domains and interaction partners. This prevented us from modeling further interaction partners of much larger macromolecular complexes (Ponce-Balbuena et al., 2018). Unfortunately, current *ab initio* prediction methods, such as AlphaFold, while performing remarkably well on core regions of proteins, are not yet able to reliably predict intrinsically disordered regions or those that fold into transient 3D structures upon interaction with other binding partners.

## 3 Methods

### 3.1 Protein-protein docking

Interactions between Na_V_1.5 (pdb: 6LQA) and K_IR_2.1 (pdb: 7ZDZ) were probed using the Fast Rotational Docking Program (FRODOCK) (Garzon et al., 2009), ClusPro2.0 (Kozakov et al., 2017), and AlpaFold-Multimer v3 (Evans et al., 2021). FRODOCK uses the rigid-body orientational sampling of a ligand molecule with respect to a fixed receptor molecule. It combines the projection of the interaction terms into 3D grid-based potentials and binding energies during complex formation. The binding energy was approximated as a correlation function consisting of van der Waals, electrostatic, and desolvation potential terms. Interaction energy minima were obtained through a fast and exhaustive rotational docking search combined with translational scanning. The program was obtained from https://chaconlab.org/modeling/frodock/. The poses were visually filtered based on their relative orientations towards each other. The relative orientation of the membrane proteins in the membrane enabled a good discrimination between plausible and implausible poses. Positions with flipped membrane orientations of the two channels with respect to each other, or with one channel interacting with the cytoplasmic or extracellular regions with the transmembrane core of the other channel, were excluded from further analysis.

With FRODOCK 5 poses fulfilled the proper membrane orientation criterion and were further inspected. From these, 3 poses where at the same interface (between DII and III), with ∼2–3 Å shifts in their relative orientation (see Supplementary Figure S4A). The pose with the largest number of contacts (as assessed with Prodigy) was chosen and shown in Figure 1.

The second rigid-body docking program was ClusPro v2.0 (Kozakov et al., 2017). It uses Piper, which is a fast Fourier transform program developed by the Vajda lab for a rigid-body global search, and employs pairwise interaction potentials. The program provides 1,000 low-energy results to the clustering program ClusPro, which then attempts to find the native site based on the underlying assumption that it will have a wide free-energy attractor with the largest number of results. The program was accessed through its web server https://cluspro.org/login.php. Similar visual filtering of poses as above was performed, and the resulting five best poses were analyzed in detail. Two poses were found at the same interface (between DII and III), while at all other interfaces only one pose was found (see Supplementary Figure S4B). The pose with the larger number of contacts (as assessed with Prodigy) was chosen and shown in Figure 1.

AlpaFold-Multimer v3 uses multiple sequence alignments (MSA) as inputs to predict the structure of protein complexes. For heteromultimers, as is the case for the Na_V_1.5-K_IR_2.1 (five independent subunits), it leverages both MSAunpaired and MSApaired as inputs. Different parameters were tested and the best results were obtained using the following parameters: mmsequs2_uniref_env, no-template mode; number of ensembles: three; number of recycles: ten; pair mode: paired; recycling early stop tolerance: 0.5; and training mode: no. Only the best of the five models obtained using these parameters was further analyzed. The model-predicted pLDDT (predicted local distance difference test) score per residue is shown in Supplementary Figure S2. The mean pLDDT score was 78.29 with an iPTM (average quality of the all interfaces of the complex) score of 0.89.

Table 3 provides an overview of the different parameters used for PPI docking by ClusPro, FRODOCK and AlphaFold multimer.

**TABLE 3:** Overview of parameters used for PPI docking.

ClusPro	FRODOCK	AlphaFold_multimer_v3
All heteroatoms including small molecule ligands, lipids and ions were removed from the protein structures before docking. Receptor definition: Na_V_1.5 α-subunit (pdb: 6LQA); Ligand definition: K_IR_2.1 (pdb: 7ZDZ), no advanced options nor restraints were used to bias docking	All heteroatoms including small molecule ligands, lipids and ions were removed from the protein structures before docking. Receptor definition: Na_V_1.5 α-subunit (pdb: 6LQA), all heteroatoms (small molecule ligand and lipids) were removed; Ligand definition: K_IR_2.1 (pdb: 7ZDZ), no advanced options nor restraints were used to bias docking	The target sequences were obtained from Uniprot, codes: Q14524-1 (SCN5A_HUMAN) including residues 1,187–1928 and P63252 · KCNJ2_HUMAN, including residues 45–387
		Model type was set to multimer_v3
		Template mode: no
		Multisequence database used: mmsequs2_uniref_env
		Pair mode: paired number of ensembles: three
		number of recycles: ten
		recycling early stop tolerance: 0.5
		training mode: no

### 3.2 Interface analysis

Interface analysis was performed using the PISA-PDBe web server (https://www.ebi.ac.uk/msd-srv/prot_int/cgi-bin/piserver) and Prodigy (Xue et al., 2016) (https://bianca.science.uu.nl/prodigy/). Both web servers enable a quick analysis of protein-protein interfaces, including the number of contacts within a certain cutoff, and simplified binding affinity predictions, which do not reflect the true energies of the complexes but are useful in quickly ranking model poses obtained from different docking programs.

## Data Availability

The original contributions presented in the study are included in the article/Supplementary Material, further inquiries can be directed to the corresponding author. The models presented in the study are publicly available. This data can be found here: https://zenodo.org/records/10677482.
