# Supplementary material for: In silico models of the macromolecular NaV1.5-KIR2.1 complex
Source: Front Physiol. 2024 Feb 26;15:1362964. doi: 10.3389/fphys.2024.1362964 (PMC10925717; doi:10.3389/fphys.2024.1362964)
Supplement: Supplementary file 1 [file DataSheet1.docx]

**Supplementary material**


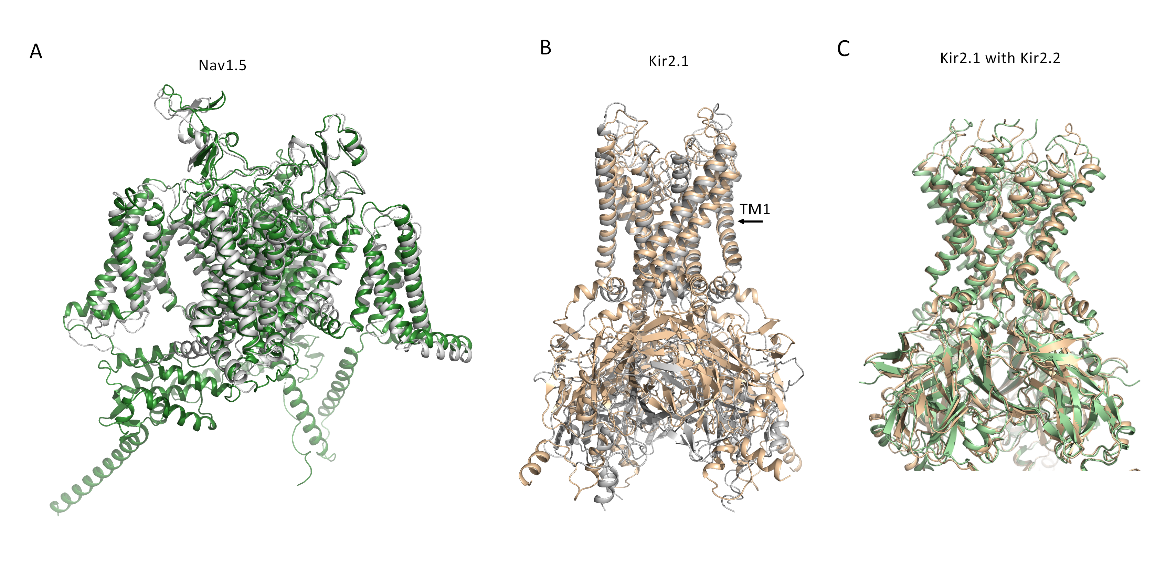


Supplemental Figure 1– A) Structural alignment between Nav1.5 cryo-EM structure, colored in grey (pdb: 6LQA) and the Alphafold generated model (dark green). B) Structural alignment between the closed state conformation of KIR2.1 (pdb: 7zdz) shown in grey and the Alphafold generated model, colored in wheat. TM1 helices in both states are virtually identical. C) Structural alignment between the open state conformation of KIR2.2 (pdb: 6M84) shown in light green and the KIR2.1 model colored in wheat.

The structural alignment was performed with Swiss-PdB-Viewer4.1.0 (Guex and Peitsch 1997). The overall RMSD is 1.5 Å over 3252 atoms, when comparing the Na_V_1.5 cryo-EM structure with the Nav1.5 coordinates modelled by Alphafold-Multimer.

The structural alignment between the K_IR_2.1 parts failed, thus the P-helical segments and SF-region only was used to obtain a more “accurate” 3D alignment. The resulting RMSD between these segments was 1.1 Å, and included 288 atoms.


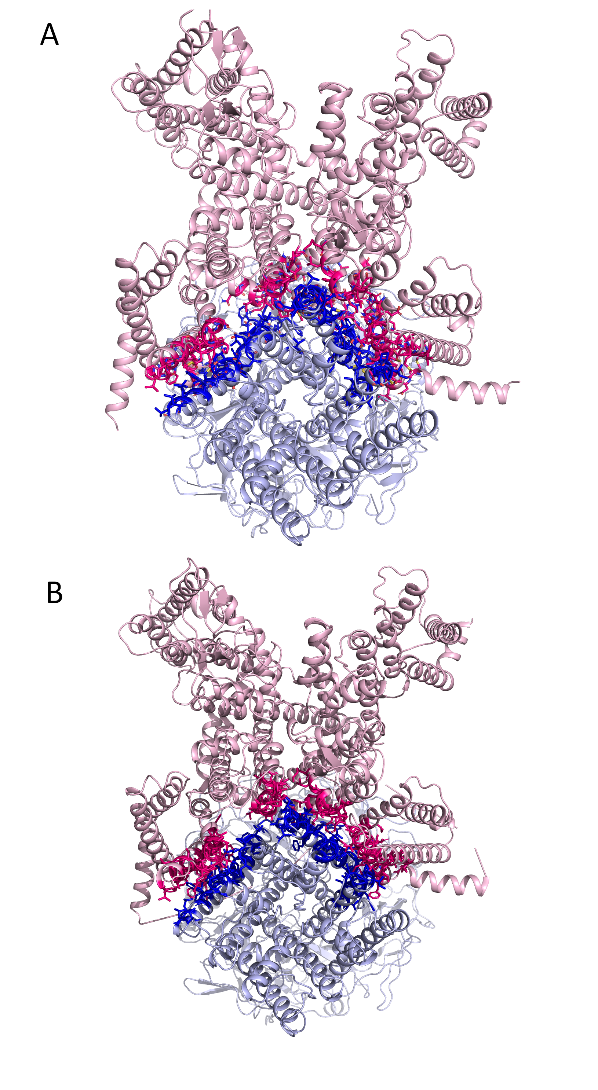


Supplemental Figure 2 – A) interface residues between Nav1.5 (pink) and KIR2.1 (blue) in the best ClusPro model are shown as sticks. B) Interface residues between Nav1.5 (pink) and KIR2.1 (blue) in the best FRODOCK model are shown as sticks. All residues within a cut-off of 5.5 Å are shown. Figure generated from PRODIGY (Xue et al. 2016) output.

Supplemental Table 1 – ClusPro interface residues (best model, 5.5 Å cut-off, interface residues as in supplemental Figure 2A), calculated with PRODIGY.

| **Residue** | **Number** | **KIR2.1 Chain** | **Residue2** | **Number2** | **Nav1.5 Chain** |
| --- | --- | --- | --- | --- | --- |
| TYR | 995 | A | THR | 1268 | B |
| ASP | 716 | A | TYR | 739 | B |
| ALA | 1018 | A | TRP | 1273 | B |
| ASN | 1054 | A | MET | 1296 | B |
| ASP | 718 | A | THR | 1723 | B |
| VAL | 690 | A | MET | 725 | B |
| VAL | 1013 | A | VAL | 1328 | B |
| ILE | 1014 | A | ALA | 1270 | B |
| PHE | 1056 | A | PRO | 1298 | B |
| ARG | 1009 | A | SER | 1333 | B |
| PHE | 445 | A | MET | 725 | B |
| LEU | 698 | A | ILE | 1343 | B |
| PHE | 441 | A | PHE | 756 | B |
| PHE | 1025 | A | TRP | 1258 | B |
| PRO | 437 | A | LEU | 749 | B |
| PRO | 437 | A | LEU | 736 | B |
| PHE | 702 | A | LEU | 1338 | B |
| ILE | 710 | A | ILE | 1749 | B |
| LEU | 713 | A | SER | 1744 | B |
| TRP | 1031 | A | VAL | 1287 | B |
| SER | 732 | A | PHE | 745 | B |
| LEU | 694 | A | SER | 821 | B |
| TRP | 1008 | A | GLY | 1329 | B |
| ARG | 1009 | A | GLY | 1329 | B |
| TRP | 1031 | A | SER | 1285 | B |
| PHE | 702 | A | LEU | 1339 | B |
| TRP | 1010 | A | GLY | 1329 | B |
| LEU | 1035 | A | ALA | 1288 | B |
| PHE | 1060 | A | LEU | 1302 | B |
| LEU | 1021 | A | PHE | 1276 | B |
| PHE | 733 | A | GLU | 737 | B |
| ARG | 1007 | A | LYS | 1479 | B |
| VAL | 449 | A | PHE | 718 | B |
| LEU | 717 | A | THR | 1723 | B |
| TRP | 1008 | A | ASN | 1472 | B |
| TRP | 1031 | A | ALA | 1294 | B |
| TRP | 687 | A | ASP | 720 | B |
| VAL | 1013 | A | ILE | 1331 | B |
| GLU | 757 | A | LYS | 1300 | B |
| ARG | 1009 | A | ASN | 1472 | B |
| LEU | 712 | A | ALA | 1407 | B |
| ASN | 731 | A | HIS | 738 | B |
| HIS | 714 | A | PRO | 1745 | B |
| LEU | 701 | A | LEU | 1346 | B |
| THR | 734 | A | LEU | 736 | B |
| PHE | 445 | A | LEU | 721 | B |
| ASP | 998 | A | LYS | 1264 | B |
| VAL | 771 | A | MET | 1335 | B |
| LEU | 701 | A | THR | 731 | B |
| TRP | 1008 | A | PRO | 1332 | B |
| ARG | 1009 | A | PRO | 1332 | B |
| TRP | 708 | A | HIS | 738 | B |
| ASP | 756 | A | PRO | 1298 | B |
| CYS | 693 | A | LEU | 721 | B |
| TRP | 1031 | A | LEU | 1283 | B |
| ILE | 760 | A | VAL | 1747 | B |
| LEU | 709 | A | ALA | 1407 | B |
| ASN | 731 | A | ASN | 740 | B |
| TRP | 1010 | A | PRO | 1332 | B |
| LYS | 721 | A | GLY | 1724 | B |
| TRP | 1031 | A | ILE | 1299 | B |
| LEU | 694 | A | MET | 725 | B |
| CYS | 705 | A | LEU | 1346 | B |
| LEU | 717 | A | LEU | 1721 | B |
| TRP | 708 | A | ASN | 1404 | B |
| PHE | 1025 | A | PHE | 1276 | B |
| ARG | 686 | A | ASP | 716 | B |
| PHE | 441 | A | CYS | 726 | B |
| ILE | 691 | A | TRP | 822 | B |
| LEU | 713 | A | ILE | 1749 | B |
| VAL | 690 | A | SER | 821 | B |
| TRP | 687 | A | LYS | 820 | B |
| TYR | 995 | A | ASN | 1269 | B |
| TRP | 1010 | A | ALA | 1326 | B |
| GLN | 670 | A | ALA | 944 | B |
| LEU | 1024 | A | ASP | 1280 | B |
| LEU | 1040 | A | GLY | 1292 | B |
| TYR | 672 | A | PRO | 823 | B |
| LEU | 1021 | A | TRP | 1273 | B |
| HIS | 714 | A | SER | 1744 | B |
| TYR | 995 | A | LYS | 1265 | B |
| LEU | 713 | A | PRO | 1745 | B |
| SER | 1055 | A | PRO | 1298 | B |
| LEU | 694 | A | LEU | 818 | B |
| LEU | 1035 | A | LEU | 1291 | B |
| PHE | 1056 | A | VAL | 1284 | B |
| ILE | 760 | A | ALA | 1746 | B |
| VAL | 444 | A | LEU | 729 | B |
| PHE | 702 | A | MET | 1335 | B |
| GLN | 670 | A | ASP | 1471 | B |
| ARG | 671 | A | GLN | 1475 | B |
| HIS | 714 | A | ALA | 1746 | B |
| PHE | 733 | A | HIS | 738 | B |
| LEU | 1035 | A | VAL | 1287 | B |
| ARG | 686 | A | PRO | 717 | B |
| VAL | 697 | A | MET | 725 | B |
| ASP | 716 | A | ASN | 1404 | B |
| PHE | 445 | A | LEU | 729 | B |
| GLN | 670 | A | LYS | 1478 | B |
| PHE | 702 | A | LEU | 1342 | B |
| PHE | 733 | A | ASN | 1404 | B |
| VAL | 690 | A | ASP | 720 | B |
| PHE | 1025 | A | TRP | 1273 | B |
| PHE | 441 | A | PHE | 733 | B |
| LEU | 709 | A | LEU | 1410 | B |
| LEU | 712 | A | ASN | 1404 | B |
| ARG | 1009 | A | GLN | 1476 | B |
| SER | 1055 | A | ILE | 1299 | B |
| LEU | 1032 | A | VAL | 1284 | B |
| LEU | 996 | A | LYS | 1264 | B |
| SER | 1022 | A | TRP | 1273 | B |
| CYS | 1028 | A | ASP | 1280 | B |
| PRO | 759 | A | LEU | 1302 | B |
| LEU | 698 | A | LEU | 1339 | B |
| TRP | 687 | A | THR | 724 | B |
| THR | 1057 | A | PRO | 1298 | B |
| VAL | 697 | A | VAL | 728 | B |
| LEU | 713 | A | ALA | 1746 | B |
| PHE | 733 | A | LEU | 732 | B |
| LEU | 1040 | A | PHE | 1293 | B |
| TYR | 995 | A | LYS | 1264 | B |
| LYS | 721 | A | PRO | 1725 | B |
| LEU | 698 | A | VAL | 728 | B |
| LEU | 709 | A | GLY | 1406 | B |
| PRO | 759 | A | PRO | 1298 | B |
| GLU | 435 | A | GLU | 744 | B |
| LEU | 1032 | A | ASP | 1280 | B |
| CYS | 436 | A | PHE | 745 | B |
| VAL | 690 | A | LEU | 721 | B |
| TRP | 708 | A | VAL | 1405 | B |
| LYS | 721 | A | THR | 1723 | B |
| TRP | 685 | A | PRO | 717 | B |
| VAL | 706 | A | LEU | 1342 | B |
| PRO | 437 | A | PHE | 745 | B |
| LEU | 694 | A | THR | 724 | B |
| ASP | 998 | A | THR | 1268 | B |
| VAL | 690 | A | PRO | 717 | B |
| ARG | 686 | A | ASP | 720 | B |
| ILE | 691 | A | SER | 821 | B |
| PHE | 1025 | A | ASP | 1280 | B |
| CYS | 693 | A | MET | 725 | B |
| PHE | 733 | A | ALA | 735 | B |
| ILE | 452 | A | PHE | 718 | B |
| LEU | 709 | A | ILE | 1749 | B |
| PHE | 1056 | A | VAL | 1281 | B |
| TRP | 1010 | A | ASN | 1325 | B |
| GLU | 757 | A | PRO | 1298 | B |
| LEU | 717 | A | PRO | 1745 | B |
| ILE | 438 | A | GLY | 752 | B |
| ALA | 719 | A | THR | 1723 | B |
| ILE | 691 | A | PRO | 823 | B |
| PRO | 759 | A | SER | 1301 | B |
| CYS | 436 | A | MET | 748 | B |
| PHE | 737 | A | LEU | 736 | B |
| ILE | 452 | A | MET | 725 | B |
| ILE | 710 | A | LEU | 1750 | B |
| ASP | 716 | A | ASP | 1403 | B |
| LEU | 694 | A | TRP | 822 | B |
| CYS | 705 | A | VAL | 1405 | B |
| ARG | 684 | A | PRO | 717 | B |
| LEU | 1012 | A | PRO | 1332 | B |
| PRO | 437 | A | MET | 748 | B |
| LEU | 1032 | A | VAL | 1287 | B |
| ILE | 438 | A | MET | 748 | B |
| TRP | 708 | A | GLY | 1406 | B |
| LEU | 701 | A | LEU | 1342 | B |
| THR | 734 | A | PHE | 745 | B |
| ILE | 710 | A | ALA | 1746 | B |
| GLU | 435 | A | PHE | 745 | B |
| PHE | 737 | A | LEU | 732 | B |
| ILE | 760 | A | LEU | 1750 | B |
| GLN | 670 | A | GLN | 1475 | B |
| PHE | 1056 | A | ILE | 1299 | B |
| LEU | 1021 | A | LEU | 1277 | B |
| PHE | 445 | A | CYS | 726 | B |
| VAL | 449 | A | MET | 725 | B |
| GLU | 757 | A | SER | 1301 | B |
| ARG | 1009 | A | PHE | 1473 | B |
| LEU | 1040 | A | LEU | 1291 | B |
| PHE | 733 | A | VAL | 1405 | B |
| TRP | 1031 | A | ALA | 1288 | B |
| CYS | 705 | A | LEU | 1342 | B |
| VAL | 697 | A | LEU | 729 | B |
| ALA | 695 | A | TRP | 822 | B |
| TYR | 672 | A | LYS | 830 | B |
| LEU | 709 | A | TYR | 1409 | B |
| LEU | 701 | A | LEU | 732 | B |
| TRP | 1008 | A | GLN | 1475 | B |
| ARG | 1009 | A | GLN | 1475 | B |
| VAL | 690 | A | THR | 724 | B |
| TRP | 1031 | A | VAL | 1284 | B |
| ILE | 452 | A | LEU | 721 | B |
| GLU | 435 | A | MET | 748 | B |
| CYS | 705 | A | GLY | 1406 | B |
| CYS | 1028 | A | VAL | 1284 | B |
| TYR | 672 | A | ASN | 826 | B |
| LEU | 698 | A | LEU | 1342 | B |
| TRP | 700 | A | LEU | 732 | B |
| LEU | 701 | A | VAL | 728 | B |
| ARG | 1009 | A | ASP | 1471 | B |
| ARG | 1009 | A | VAL | 1328 | B |
| LEU | 1035 | A | GLY | 1292 | B |
| LEU | 713 | A | LEU | 1721 | B |
| GLU | 757 | A | GLY | 1297 | B |
| TRP | 1010 | A | VAL | 1328 | B |
| LEU | 717 | A | LYS | 1399 | B |
| PHE | 733 | A | LEU | 736 | B |
| ASP | 434 | A | PHE | 745 | B |
| PHE | 733 | A | GLY | 1406 | B |
| ARG | 686 | A | MET | 715 | B |
| PHE | 441 | A | LEU | 729 | B |
| LEU | 701 | A | ALA | 735 | B |
| PRO | 437 | A | PHE | 733 | B |
| LEU | 712 | A | GLY | 1406 | B |
| ARG | 1009 | A | ALA | 1330 | B |
| ASN | 1054 | A | PRO | 1298 | B |
| PHE | 445 | A | THR | 722 | B |
| ILE | 438 | A | PHE | 733 | B |
| LEU | 1017 | A | ALA | 1270 | B |
| LEU | 694 | A | VAL | 728 | B |
| LEU | 698 | A | LEU | 818 | B |
| ILE | 438 | A | VAL | 751 | B |
| ASP | 1005 | A | LYS | 1479 | B |
| TRP | 687 | A | SER | 821 | B |
| PHE | 1056 | A | LEU | 1302 | B |
| LEU | 1017 | A | TRP | 1273 | B |
| LEU | 1024 | A | LEU | 1277 | B |
| SER | 732 | A | HIS | 738 | B |
| ILE | 448 | A | MET | 725 | B |
| LEU | 1036 | A | LEU | 1291 | B |

Supplemental Table 2 - FRODOCK interface residues (best model, 5.5 Å cut-off, interface residues as in supplemental Figure 2B), calculated with PRODIGY.

| **Residue** | **Number** | **KIR2.1 Chain** | **Residue2** | **Number2** | **Nav1.5 Chain** |
| --- | --- | --- | --- | --- | --- |
| TRP | 731 | A | ASN | 774 | B |
| THR | 759 | A | PHE | 358 | B |
| LEU | 717 | A | LEU | 431 | B |
| LEU | 724 | A | LEU | 345 | B |
| THR | 1093 | A | ILE | 669 | B |
| LEU | 732 | A | GLY | 776 | B |
| PHE | 725 | A | MET | 705 | B |
| TRP | 1065 | A | ALA | 658 | B |
| LEU | 712 | A | PRO | 330 | B |
| LEU | 736 | A | ILE | 1119 | B |
| ILE | 453 | A | PHE | 369 | B |
| LEU | 1051 | A | TRP | 641 | B |
| THR | 759 | A | LEU | 349 | B |
| PHE | 1059 | A | PHE | 646 | B |
| LEU | 1055 | A | PHE | 637 | B |
| PHE | 1092 | A | LEU | 672 | B |
| LYS | 745 | A | THR | 1093 | B |
| ASN | 1090 | A | PRO | 668 | B |
| ILE | 453 | A | GLY | 365 | B |
| LEU | 732 | A | LEU | 780 | B |
| PHE | 789 | A | TRP | 643 | B |
| PHE | 758 | A | LEU | 349 | B |
| TRP | 1065 | A | VAL | 654 | B |
| PHE | 725 | A | LEU | 712 | B |
| LEU | 1051 | A | PHE | 637 | B |
| LEU | 741 | A | LYS | 769 | B |
| MET | 457 | A | LEU | 342 | B |
| LEU | 724 | A | LEU | 712 | B |
| LEU | 735 | A | VAL | 775 | B |
| PHE | 460 | A | MET | 338 | B |
| ASP | 742 | A | THR | 1093 | B |
| VAL | 455 | A | LEU | 342 | B |
| VAL | 729 | A | LEU | 712 | B |
| PHE | 460 | A | LEU | 334 | B |
| SER | 1078 | A | MET | 666 | B |
| LEU | 1058 | A | PHE | 646 | B |
| SER | 1091 | A | ILE | 669 | B |
| LEU | 736 | A | LEU | 1120 | B |
| ALA | 718 | A | TRP | 435 | B |
| THR | 759 | A | LEU | 345 | B |
| PHE | 456 | A | ILE | 340 | B |
| LEU | 724 | A | VAL | 341 | B |
| LEU | 1051 | A | ALA | 640 | B |
| PHE | 758 | A | LEU | 345 | B |
| PRO | 452 | A | PHE | 346 | B |
| PHE | 456 | A | PHE | 369 | B |
| LEU | 717 | A | MET | 338 | B |
| PRO | 785 | A | SER | 671 | B |
| TYR | 695 | A | PRO | 436 | B |
| SER | 1091 | A | ALA | 658 | B |
| GLU | 782 | A | ILE | 669 | B |
| ALA | 1052 | A | TRP | 643 | B |
| CYS | 716 | A | MET | 338 | B |
| LEU | 1055 | A | LEU | 647 | B |
| LEU | 721 | A | LEU | 709 | B |
| PHE | 758 | A | VAL | 775 | B |
| LEU | 1069 | A | GLY | 662 | B |
| LEU | 736 | A | GLY | 1118 | B |
| PRO | 452 | A | LEU | 342 | B |
| ILE | 714 | A | LEU | 334 | B |
| ARG | 1043 | A | GLY | 699 | B |
| ASP | 781 | A | PRO | 668 | B |
| ARG | 709 | A | PRO | 330 | B |
| LEU | 736 | A | GLY | 776 | B |
| PHE | 726 | A | MET | 705 | B |
| VAL | 713 | A | LEU | 334 | B |
| LEU | 724 | A | THR | 344 | B |
| PHE | 456 | A | ASN | 343 | B |
| LEU | 741 | A | ILE | 1090 | B |
| TRP | 1065 | A | VAL | 657 | B |
| PHE | 1092 | A | ILE | 669 | B |
| PRO | 452 | A | LEU | 349 | B |
| ILE | 453 | A | VAL | 368 | B |
| LEU | 741 | A | ILE | 1119 | B |
| LEU | 736 | A | LEU | 780 | B |
| LEU | 1051 | A | TYR | 636 | B |
| PHE | 456 | A | CYS | 339 | B |
| TRP | 731 | A | VAL | 775 | B |
| ILE | 786 | A | ALA | 1116 | B |
| THR | 1093 | A | LEU | 672 | B |
| ILE | 1048 | A | PHE | 637 | B |
| ASP | 740 | A | PRO | 1115 | B |
| ARG | 1043 | A | LYS | 849 | B |
| ILE | 453 | A | PHE | 346 | B |
| ASP | 781 | A | SER | 671 | B |
| PHE | 1096 | A | VAL | 651 | B |
| CYS | 728 | A | LEU | 712 | B |
| GLU | 782 | A | GLY | 667 | B |
| PHE | 1059 | A | TRP | 628 | B |
| THR | 1093 | A | PRO | 668 | B |
| LEU | 712 | A | LEU | 334 | B |
| VAL | 1054 | A | TRP | 643 | B |
| PHE | 762 | A | LEU | 349 | B |
| LEU | 1075 | A | GLY | 662 | B |
| LEU | 741 | A | THR | 1093 | B |
| ILE | 453 | A | LEU | 342 | B |
| LEU | 735 | A | ILE | 1119 | B |
| LEU | 736 | A | THR | 1123 | B |
| PHE | 1096 | A | LEU | 647 | B |
| ALA | 734 | A | ALA | 1116 | B |
| ARG | 709 | A | MET | 328 | B |
| LEU | 717 | A | VAL | 341 | B |
| LEU | 741 | A | LEU | 1091 | B |
| ALA | 1052 | A | THR | 638 | B |
| TRP | 1065 | A | LEU | 653 | B |
| LEU | 1058 | A | ASP | 650 | B |
| LEU | 741 | A | PRO | 1115 | B |
| ILE | 1048 | A | ALA | 640 | B |
| LEU | 1058 | A | TRP | 643 | B |
| LEU | 736 | A | VAL | 1117 | B |
| TYR | 1029 | A | LYS | 634 | B |
| LEU | 1055 | A | TRP | 643 | B |
| LEU | 732 | A | VAL | 775 | B |
| LEU | 717 | A | THR | 337 | B |
| GLU | 782 | A | LYS | 670 | B |
| GLY | 739 | A | PRO | 1115 | B |
| PHE | 456 | A | THR | 335 | B |
| ILE | 463 | A | MET | 338 | B |
| LEU | 721 | A | VAL | 341 | B |
| LEU | 717 | A | SER | 434 | B |
| LEU | 721 | A | LEU | 431 | B |
| LEU | 1051 | A | TRP | 643 | B |
| LEU | 1069 | A | LEU | 661 | B |
| PHE | 762 | A | LEU | 345 | B |
| ILE | 714 | A | SER | 434 | B |
| SER | 1091 | A | PRO | 668 | B |
| VAL | 713 | A | THR | 337 | B |
| ILE | 463 | A | LEU | 334 | B |
| TRP | 710 | A | THR | 337 | B |
| TRP | 1065 | A | LEU | 656 | B |
| GLY | 739 | A | ALA | 1116 | B |
| VAL | 713 | A | SER | 434 | B |
| PHE | 725 | A | LEU | 708 | B |
| TRP | 710 | A | SER | 434 | B |
| LEU | 736 | A | LEU | 1091 | B |
| LEU | 1069 | A | VAL | 657 | B |
| GLU | 449 | A | PHE | 358 | B |
| ASN | 1090 | A | MET | 666 | B |
| ILE | 733 | A | VAL | 1117 | B |
| LEU | 1051 | A | ASN | 639 | B |
| PHE | 456 | A | LEU | 342 | B |
| LEU | 1051 | A | LEU | 644 | B |
| LEU | 736 | A | PRO | 1115 | B |
| ASP | 742 | A | PRO | 1115 | B |
| PRO | 785 | A | LEU | 675 | B |
| LEU | 1055 | A | PHE | 646 | B |
| PHE | 1092 | A | ASP | 650 | B |
| LEU | 735 | A | ALA | 777 | B |
| GLU | 782 | A | PRO | 668 | B |
| ASP | 740 | A | ASN | 774 | B |
| LEU | 721 | A | ILE | 713 | B |
| LEU | 741 | A | ASN | 1092 | B |
| PHE | 460 | A | ILE | 336 | B |
| CYS | 1050 | A | TRP | 643 | B |
| LEU | 736 | A | ALA | 1116 | B |
| LEU | 717 | A | TRP | 435 | B |
| LEU | 1055 | A | CYS | 642 | B |
| LEU | 735 | A | GLY | 776 | B |
| VAL | 713 | A | ASP | 333 | B |
| TRP | 710 | A | ASP | 333 | B |
| VAL | 1047 | A | VAL | 698 | B |
| GLU | 449 | A | MET | 361 | B |
| LEU | 1075 | A | LEU | 661 | B |
| ILE | 733 | A | LEU | 1120 | B |
| LEU | 1051 | A | CYS | 642 | B |
| VAL | 720 | A | VAL | 341 | B |
| ASN | 1090 | A | ILE | 669 | B |
| PHE | 1096 | A | LEU | 672 | B |
| GLU | 782 | A | SER | 671 | B |
| LEU | 712 | A | PHE | 331 | B |
| LEU | 1055 | A | TYR | 636 | B |
| PHE | 456 | A | MET | 338 | B |
| LEU | 721 | A | TRP | 435 | B |
| ILE | 467 | A | LEU | 334 | B |
| PHE | 726 | A | LEU | 709 | B |
| LEU | 1051 | A | THR | 638 | B |
| PHE | 1092 | A | VAL | 651 | B |
| PHE | 460 | A | CYS | 339 | B |
| VAL | 464 | A | PHE | 331 | B |
| ILE | 733 | A | ALA | 1116 | B |
| TRP | 708 | A | PRO | 330 | B |
| ILE | 786 | A | VAL | 1117 | B |
| LEU | 732 | A | ILE | 1119 | B |
| VAL | 459 | A | MET | 338 | B |
| LEU | 735 | A | ASN | 774 | B |
| ALA | 1052 | A | PHE | 637 | B |
| LEU | 1058 | A | LEU | 647 | B |
| ASP | 448 | A | PHE | 358 | B |
| ALA | 760 | A | LEU | 349 | B |
| TRP | 731 | A | ALA | 777 | B |
| LEU | 717 | A | LEU | 334 | B |
| THR | 759 | A | ALA | 348 | B |
| CYS | 716 | A | LEU | 334 | B |
| PHE | 1092 | A | VAL | 654 | B |
| CYS | 1062 | A | ASP | 650 | B |
| PHE | 758 | A | ALA | 348 | B |
| LEU | 736 | A | SER | 1114 | B |
| ILE | 786 | A | LEU | 1120 | B |
| LEU | 732 | A | TYR | 779 | B |
| CYS | 728 | A | LEU | 716 | B |
| TRP | 731 | A | GLY | 776 | B |
| VAL | 720 | A | MET | 338 | B |
| PHE | 725 | A | LEU | 709 | B |
| SER | 757 | A | PHE | 358 | B |
| LYS | 745 | A | GLY | 1094 | B |
| PHE | 460 | A | THR | 335 | B |
| PHE | 1096 | A | VAL | 654 | B |
| TRP | 1065 | A | SER | 655 | B |
| LEU | 724 | A | LEU | 709 | B |
| VAL | 713 | A | PRO | 330 | B |
| PHE | 1059 | A | PHE | 637 | B |
| TRP | 710 | A | PRO | 330 | B |
| SER | 757 | A | LEU | 349 | B |
| TRP | 710 | A | LYS | 433 | B |
| PHE | 456 | A | VAL | 341 | B |
| LEU | 732 | A | LEU | 712 | B |
| SER | 722 | A | LEU | 709 | B |
| LYS | 745 | A | PRO | 1095 | B |
| LEU | 732 | A | ALA | 777 | B |
| LEU | 735 | A | PRO | 1115 | B |
| PRO | 452 | A | MET | 361 | B |
| CYS | 1062 | A | PHE | 646 | B |
| PHE | 460 | A | LEU | 342 | B |
| PHE | 1059 | A | PHE | 633 | B |
| VAL | 1047 | A | ALA | 640 | B |
| ILE | 1048 | A | THR | 638 | B |
| ASN | 756 | A | ASN | 774 | B |


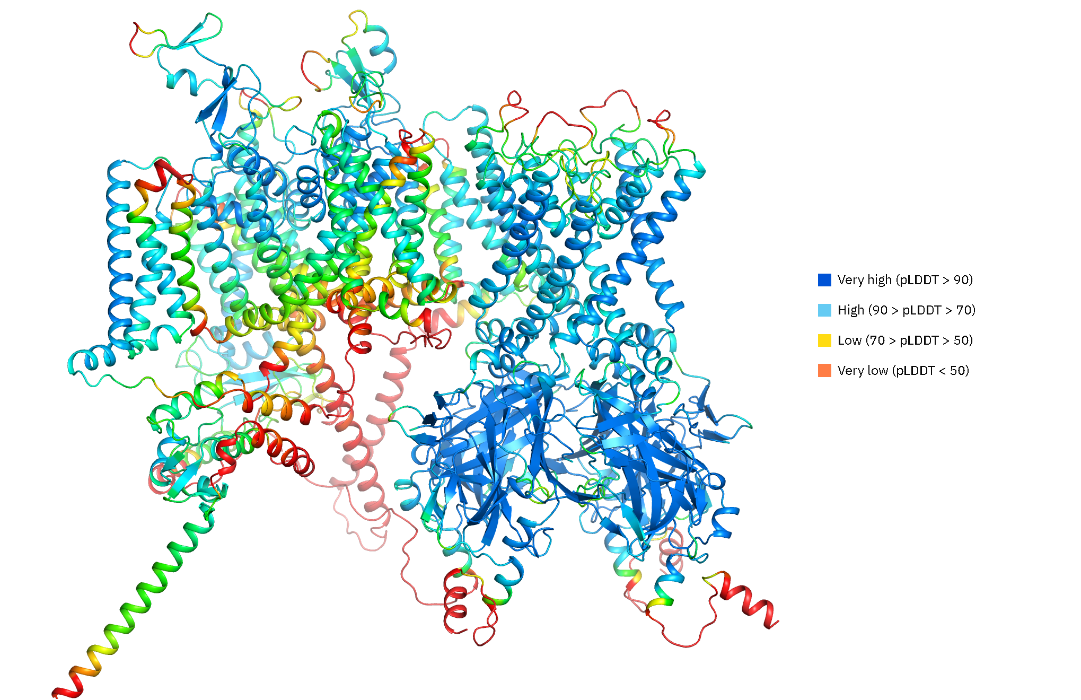


Supplemental Figure 3 – pLDDT score of best Alphafold-Multimer model


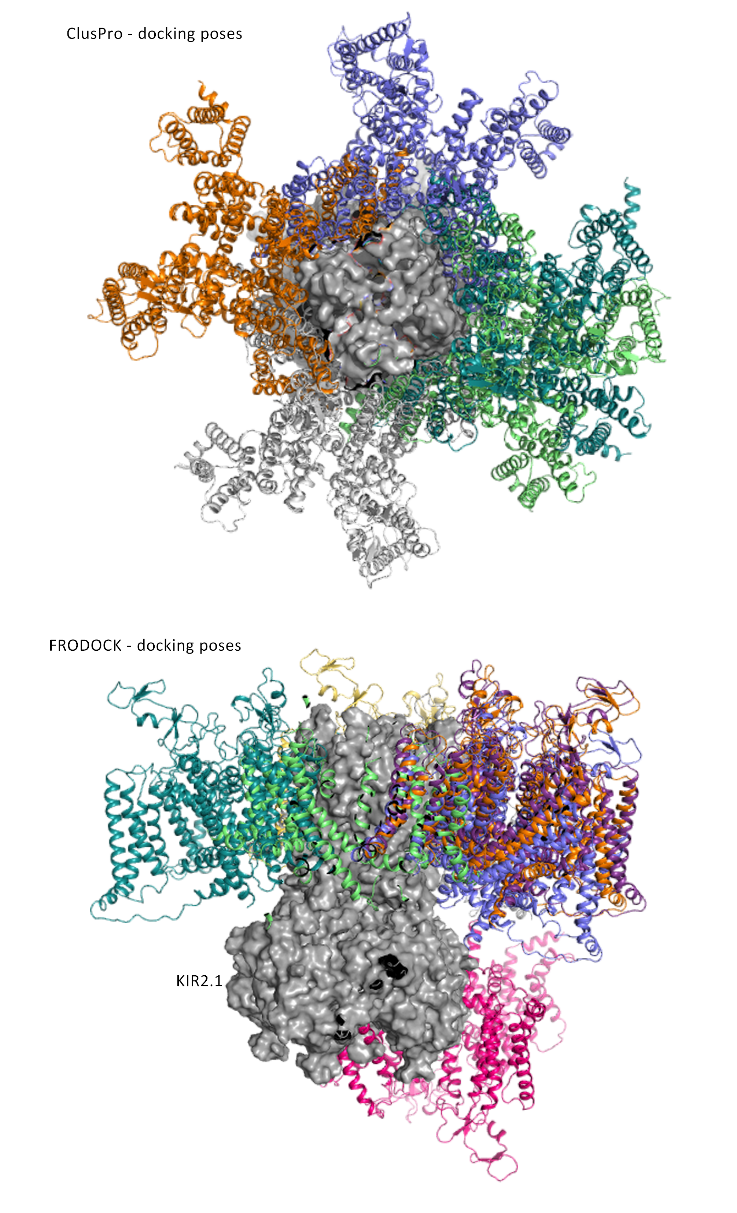


Supplemental Figure 4 – selected poses from rigid protein-protein docking. Top panel: the best 5 poses from ClusPro are shown, with the Kir2.1 channel shown as grey surface and the predicted Nav1.5 poses shown as cartoons, coloured by prediction model. Bottom panel: 7 docking poses are shown, using the same color and representation scheme as above. The pink Nav1.5 orientation shows an example of implausible membrane orientation, with the Nav1.5 TM core predicted to interact with the cytoplasmic domain of K_IR_2.1.

**References**

Guex, Nicolas, and Manuel C. Peitsch. 1997. ‘SWISS‐MODEL and the Swiss‐Pdb Viewer: An Environment for Comparative Protein Modeling’. *ELECTROPHORESIS* 18 (15): 2714–23. https://doi.org/10.1002/elps.1150181505.

Xue, Li C., João Pglm Rodrigues, Panagiotis L. Kastritis, Alexandre Mjj Bonvin, and Anna Vangone. 2016. ‘PRODIGY: A Web Server for Predicting the Binding Affinity of Protein–Protein Complexes’. *Bioinformatics* 32 (23): 3676–78. https://doi.org/10.1093/bioinformatics/btw514.
